# SRL pathogenicity island contributes to the metabolism of D-aspartate via an aspartate racemase in *Shigella flexneri* YSH6000

**DOI:** 10.1371/journal.pone.0228178

**Published:** 2020-01-24

**Authors:** Tania Henríquez, Juan Carlos Salazar, Massimiliano Marvasi, Ajit Shah, Gino Corsini, Cecilia S. Toro

**Affiliations:** 1 Programa de Microbiología y Micología, ICBM, Facultad de Medicina, Universidad de Chile, Santiago, Chile; 2 Biozentrum, Ludwig-Maximilians-Universität München, Martinsried, Germany; 3 Università degli Studi di Firenze, Firenze, Italy; 4 Middlesex University London, The Burroughs, London, United Kingdom; 5 Instituto de Ciencias Biomédicas, Universidad Autónoma de Chile, Santiago, Chile; University of Padova, Medical School, ITALY

## Abstract

In recent years, multidrug resistance of *Shigella* strains associated with genetic elements like pathogenicity islands, have become a public health problem. The *Shigella* resistance locus pathogenicity island (SRL PAI) of *S*. *flexneri* 2a harbors a 16Kbp region that contributes to the multidrug resistance phenotype. However, there is not much information about other functions such as metabolic, physiologic or ecological ones. For that, wild type *S*. *flexneri* YSH6000 strain, and its spontaneous SRL PAI mutant, 1363, were used to study the contribution of the island in different growth conditions. Interestingly, when both strains were compared by the Phenotype Microarrays, the ability to metabolize D-aspartic acid as a carbon source was detected in the wild type strain but not in the mutant. When D-aspartate was added to minimal medium with other carbon sources such as mannose or mannitol, the SRL PAI-positive strain was able to metabolize it, while the SRL PAI-negative strain did not. In order to identify the genetic elements responsible for this phenotype, a bioinformatic analysis was performed and two genes belonging to SRL PAI were found: *orf8*, coding for a putative aspartate racemase, and *orf9*, coding for a transporter. Thus, it was possible to measure, by an indirect analysis of racemization activity in minimal medium supplemented only with D-aspartate, that YSH6000 strain was able to transform the D-form into L-, while the mutant was impaired to do it. When the *orf8-orf9* region from SRL island was transformed into *S*. *flexneri* and *S*. *sonnei* SRL PAI-negative strains, the phenotype was restored. Although, when single genes were cloned into plasmids, no complementation was observed. Our results strongly suggest that the aspartate racemase and the transporter encoded in the SRL pathogenicity island are important for bacterial survival in environments rich in D-aspartate.

## Introduction

*Shigella* spp. is the causative agent of shigellosis, a worldwide disease common in children under the age of five [1–3]. In recent years, multidrug resistance (MDR) of several *Shigella* strains has become a public health problem [4, 5]. Among genetic elements associated to MDR, resistance genes harbored in the SRL pathogenicity island (SRL PAI) were identified as contributors to the resistance phenotype [6]. SRL PAI is a 66 kbp element that contains a 16 kbp SRL region (*Shigella* Resistance Locus), which codifies resistance to streptomycin (*aadA1*), ampicillin (*oxa*-1), chloramphenicol (*cat*) and tetracycline (*tetB*) genes [6–8]. Few genes inside the SRL island have been characterized beside the resistance genes: *int*, *rox*, and *fec* genes encoding for the ferric dicitrate transport system (Fec) [6, 7]. The Fec system seems important in ferric metabolism, however it is not essential during bacterial growth under iron limited conditions (probably due to the presence of other iron carriers) [6].

However, when different SRL PAI features have been searched, not much information is available and the role of this island in *Shigella* survival and replication inside or outside the host is not known. The primary host and natural reservoir known for *Shigella* spp. is the human gastrointestinal tract; however, it can survive in different environments: *S*. *flexneri* survival has been detected in 0.9% NaCl solution and distilled water at about 5°C for 87 and 83 days, respectively [9], and *S*. *sonnei* can survive on the surface of a lettuce for 3 days at 5°C [10]. Some studies indicate that *Shigella* spp. is capable of surviving in conditions that could be considered extreme for other enterobacteria, for example, growth at pH 2.5 [11]. This resistance can be related to the extreme environmental condition that bacteria could face in order to survive and multiply inside and outside the host. In particular, the resistance to acidic pH would be akin to passing through the human stomach (pH<3). Other important feature of *Shigella* is that it can survive within macrophages [12]. Though, to date, the mechanisms and genes involved to cope with these conditions have not been described.

Recently, our attention has been focused on analyzing other functions of the SRL PAI beyond antibiotic resistance. Altogether, 59 open reading frames (ORF) were identified within the SRL PAI, most of them are only putative [6]. Interestingly, the comparison of the SRL PAI positive-strain, YSH6000, and its isogenic mutant, 1363, showed a difference in the ability to metabolize D-aspartate (D-Asp). When protein similarity was inferred into *S*. *flexneri* SRL PAI, a putative aspartate racemase was identified, followed by a transporter associated with the aspartic acid metabolism (*orf8* and *orf9*, respectively). The activity of these genes and their role in adverse conditions of growth, such as in presence of high concentration of D-Asp, were further investigated.

## Materials and methods

### Bacterial strains and culture media

*Shigella flexneri 2a* YSH6000 and *S*. *flexneri 2a* 1363 (with a spontaneous deletion of the SRL PAI) [6] were kindly provided by Dr. Ben Adler. *Shigella sonnei* 566 wild type strain without the SRL PAI was used as a control. A complete list of strains and plasmids used in this study is available in S1 Table. All the strains were cultured in Lysogeny Broth (LB) medium and maintained as a frozen glycerol stock.

When D-Asp was used, *Shigella* was grown in minimal medium containing 1X Minimal Salt (Invitrogen, Carlsbad, US) with 2 mM MgSO_4_, 45 μM methionine, 12.5 μM nicotinic acid and 30 mM D-Asp (all reagents are from Sigma-Aldrich, St. Louis, US). Recombinant strains were grown in the presence of gentamicin (10 μg mL^-1^), when appropriate. For growth experiments using recombinant strains, optimized minimal medium containing M9 salts 1X, MgSO_4_ (2mM), CaCl_2_ (0.1mM), trace elements (134 μM EDTA, 31 μM FeCl_3_, 6.2 μM ZnCl_2_, 0.76 μM CuCl_2_, 0.42 μMCoCl_2_, 1.62 μM H_3_Bo_3_, 81 nM MnCl_2_), biotin (1 μg mL^-1^), thiamine (30 μM), methionine (10 μg mL^-1^), cysteine (10 μg mL^-1^), nicotinic acid (10 μg mL^-1^), tryptophan (20 μg mL^-1^), gentamicin (2 μg mL^-1^), IPTG (0.5 mM) and L- or D- aspartic acid (25 mM) was used.

### Complementation of the orf8-orf9 region and of each single gene in SRL PAI negative strains

Gentamicin was used as an additional selector marker since the SRL PAI already confers resistance to ampicillin, streptomycin, tetracycline and chloramphenicol. Therefore, pUC18 was modified with a gentamicin resistance (GR) cassette to select recombinant strains. To that end, GR cassette from strain TP997 (Addgene, Cambridge, Massachusetts, USA) was amplified using primers: P1-5’CGAATCCATGTGGGAGTTTA3’; P2-5’TTAGGTGGCGGTACTTGGGT3’, as described by Poteete *et al*. [13]. Then, GR cassette was cloned into pUC18 using the restriction enzyme *Ssp*I (NEB, Ipswich, USA) and ligated with T4 ligase (Invitrogen, Carlsbad, US) producing plasmid pUC18G. Secondly, region *orf8-orf9* (including the putative promoter of *orf8*, identified by BPROM software) was amplified from *S*. *flexneri* YSH6000 using primers Fw 5’CCGGAATTCAAGCGGAATGTTCCATAGCTG3’ and Rev 5’CGCGGATCCGGTATCAAAAAGCGATAGATGCAG3’ and KAPA HiFi enzyme (Kapa Biosystems). Then, it was cloned into the polylinker site of pUC18G after its digestion with *Sma*I. The resulting pUC18G *orf8-orf9* and pUC18G (empty vector) were electroporated into *S*. *flexneri* 1363 and *S*. *sonnei* 566 strains, producing complemented strains (1363 pUC18G *orf8-orf9* and 566 pUC18G *orf8-orf9*), and control strains with the empty plasmid (1363 pUC18G and 566 pUC18G) respectively. Cloning of single genes was achieve by using pUC18G as template for PCR using the primers pUC18F (5’-AGCTGTTTCCTGTGTGAAA-3’) and pUC18 Hind (5’-GGCATGCAAGCTTGGCACT-3’). With these primers, the modified pUC18 was amplified without the ATG of the *lac*Z gene, allowing the cloning of the *orf8* or *orf9* under the control of the *tac* promoter. In parallel, *orf8* was amplified from YSH6000 using the primers Fw 5’-ATGGGGCCGGCAGCTAC-3 and Rev 5’-GCGAATAAGCTTCTGGCTGCG-3’, while *orf9* was amplified using Fw 5’-ATGGTTTGGCTGGAAATT-3’ and Rev 5’-ATGTAAGCTTGTGCTAAATA-3’. Then, the vector and insert were digested with *Hind*III (NEB) and ligated using T4 DNA ligase (NEB) according to manufacturer´s instructions. The resulting plasmids pUC18G *orf8* and pUC18G *orf9* were then electroporated into *S*. *flexneri* 1363. As an additional control for optimized minimal medium, YSH6000 was electroporated with pUC18G (empty vector).

### Biochemical characterization

*S*. *flexneri* YSH6000 and *S*. *flexneri* 1363 were phenotypically characterized by microbial identification system of VITEK 2 compact (Biomerieux) according to manufacturers’ instructions. For this purpose, Gram Negative (GN) card was used. Further biochemical characterization was performed using Biolog (Biolog, Hayward, California, USA) through Phenotype Microarray (PM) assay [14] using panels one to eight. PM Biolog technology allowed us to analyze ~750 cellular phenotypes (including C, N, P, and S metabolism pathways). Each strain was tested in duplicate. Succinate was used as carbon sources for Biolog PM panels three to eight as per user’s manual recommendations. Nicotinic acid was used to cover auxotrophic requirement in plates PM one to PM eight. The redox chemistry used in PM Biolog employs cell respiration by reducing a tetrazolium dye leading to a color change. The plates were incubated at 37°C and change of color of the tetrazolium dye was measured every 15 min for 48 h.

### Bioinformatics analysis

*S*. *flexneri* YSH6000 SRL PAI was analyzed using the GenBank sequence previously deposited in 2001 (accession number AF326777.3) [6]. Nucleotide and Protein BLASTs were used to analyze putative ORFs. The analysis of putative promoters was performed using BPROM (Softberry) tool.

### Triphenyl tetrazolium chloride (TTC) reduction in presence of D-aspartic acid and mannose or mannitol as carbon source

To measure respiration of the bacteria in presence of D-Asp (metabolic activity), 1 mL of overnight LB culture of all strains was collected (15 000 rcf for 3 minutes) and washed three times with Phosphate Saline Buffer (PBS) 1X to remove medium residuals. Subsequently, pellets were suspended in 1 mL of D-Asp Minimal Medium containing 1X Minimal Salt (Invitrogen, Carlsbad, US) with 2 mM MgSO_4_, 45 μM methionine, 12.5 μM nicotinic acid and 0.01% triphenyl tetrazolium chloride (TTC). When needed, D-Asp was used at 0.01%. 40 mM mannose or 40 mM mannitol were used as carbon source. Suspensions were normalized using a Thermo Scientific, Helios Epsilon model, spectrophotometer and cultures were incubated for 24 h at 37°C and 115 rpm. After the incubation, 1 ml of cells was centrifuged (3 minutes at 15 000 rcf) and the supernatant was used to measure TTC reduction (reflecting respiration). Final absorbance (using a spectrophotometer set at 490 nm [15]) minus initial absorbance was calculated.

### Quantification of D- and L-aspartic acid using HPLC/MS-MS

1 mL of each overnight cultures in LB was washed three times with PBS and then suspended in Minimal Medium containing 30 mg mL^-1^ of D-Asp. Suspensions were incubated in a shaker for 24 h at 37°C and 115 rpm. After incubation, suspensions were filtered using a 0.20 μm polyvinylidene difluoride syringe filters (Fisher Scientific), and then stored at -80°C for liquid chromatography–mass spectrometry/mass spectrometry (LC-MS/MS) analysis. A Jasco (Chelmsford, UK) HPLC system composed of two X-LC 3085 PU pumps, a X-LC 3080DG degasser, a X-LC 3190MX mixer, a CO-2067 intelligent column oven set at 25°C and a Waters 2777 (Elstree, UK) sample manager set at 4°C. Separation was performed using a mixture of water and methanol (30:70, v/v) containing 0.1% formic acid at a flow rate of 0.21 mL min^-1^ and a 250 x 2.0 mm I.D. Chirobiotic Tag 5 μm chiral column (Sigma Aldrich, Poole, UK). The separated analytes were detected using an API3000 triple quadrupole mass spectrometer (Sciex, Warrington, UK) equipped with an electrospray interface and operated in positive ion mode. The molecular reaction monitoring (MRM) transitions used for detection of aspartate were *m/z* 133.96 → 74 and 133.96 → 88 with unit resolution. The delay time was set to 200 msec. The optimum declustering, focusing and entrance potential were found to be 34, 90 and 10 V respectively. The collision energy, collision cell exit potential, ion spray voltage and source temperature were set at 21 V, 13 V, 5000 V and 300°C respectively. In minimal medium, the peak for L-Asp was observed at 5.32 min and for D-Asp at 7.81 min.

### Quantification of bacterial growth

For the measurement of growth after overnight incubation (16h), optimized minimal medium with D-Asp was used. To that end, each strain transformed with a plasmid was grown in LB plus gentamicin (10 μg mL^-1^) until an OD ~1.8, centrifuged (3 minutes at 15 000 rcf), washed three times with PBS 1X, and then used to start a new culture with an OD ~0.2. After 16 hours of incubation at 37°C with continuous shaking (140 rpm), OD600 was measured and final OD minus initial OD was calculated.

For growth curves, cells were prepared in the same way described above and used to inoculate minimal medium with D-Asp or L-Asp. Incubation was performed at 37°C with continuous shaking (140 rpm). Each hour, 0.5 ml of culture was taken and measured at 600 nm.

### Statistical analysis

The statistical software JMP (SAS) package and Prism 8 were used to infer the one-way ANOVA analysis (p<0.05) and T-test. Tukey means separation analysis was performed in order to group the means.

## Results

### SRL PAI allows *S*. *flexneri* YSH6000 strain to metabolize D-aspartic acid

In order to identify the contribution of the SRL pathogenicity island (SRL PAI) to main metabolic routes, *S*. *flexneri* 2a YSH6000 and its isogenic mutant, *S*. *flexneri* 2a 1363 (spontaneous excision of the SRL PAI) were inoculated in Vitek 2 Compact GN cards. No differences were observed when 47 biochemical tests were used (S2 Table). A further comparison was performed through Phenotype MicroArrays (PMs) by carrying out a functional phenotypic screening of about 800 metabolites. Interestingly, the major difference observed in the screening indicated that the SLR PAI-negative strain, *S*. *flexneri* 2a 1363, lost the ability to metabolize the D-Asp (Table 1). PM analysis results did not show significant differences in the metabolism of other D-amino acids between both strains, suggesting a specific effect. This strain also gained the capability to metabolize D-raffinose (S3 Table). Since the ability to metabolize D-amino acids seems to be important for bacterial survival in the environment [16], respiration in presence of D-Asp was selected for further characterization.

**Table 1:** Phenotype microarray comparison between *S*. *flexneri* 2a 1363 against *S*. *flexneri* 2a YSH6000.

Difference in respiration*	Test	Phenotype	Mode of action
54	D-raffinose	Gained	C-Source; carbohydrate
-109	D-aspartic acid	Lost	C-Source; amino acid

* The value represents a comparison of the difference in the average height of the respiration curve of *S*. *flexneri* 2a 1363 versus *S*. *flexneri* YSH6000

D- amino acids might have a great impact on bacterial communities. Thus, the presence of D- amino acids have been reported to influence physiological processes such as biofilm formation, sporulation and peptidoglycan remodeling [17–18].

**Fig 1:**
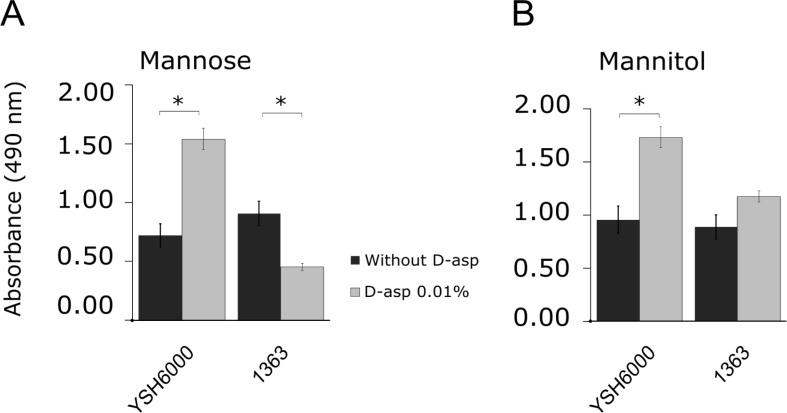
Effect of D-aspartic acid on bacterial metabolism in presence of mannose or mannitol as carbon source. Reference strains were grown in *Shigella* Minimal Medium with mannose (A) or mannitol (B) as carbon source supplemented with D-Asp at 0.01% w/v. Bars show the change on redox indicator (TTC) at 24 h of incubation (final absorbance minus initial absorbance). T-test was performed and significant results are represented by asterisks (* = *p*<0.05). Error bars represent standard deviation.

On the other hand, D- amino acids toxicity has also been reported, such as a pioneering study by Eisenstadt and coworkers which showed the detrimental effect of D-Asp on bacterial growth [19–20]. To determine whether SRL PAI has positive impact over bacteria growing in the presence of D-Asp, we measured metabolic activity of strains by using other carbon sources plus D-Asp. We selected mannose and mannitol, two sugars that were previously identified by Vitek 2 Compact as preferred carbon sources for growth. When 0.01% D-Asp was added, *S*. *flexneri* YSH6000 had a higher respiration rate (measured with the redox indicator TTC) in mannose minimal medium compared to the media without D-Asp. On the contrary, *S*. *flexneri* 1363 strain had a significant decrease in growth in mannose minimal medium when it was supplemented with D-Asp (Fig 1A). Similar findings were obtained in presence of mannitol for YSH6000 strain (Fig 1B), suggesting the toxicity of the D- form in such growth conditions and the role of SRL PAI for detoxification.

### A putative aspartate racemase is coded by *orf8*

Bioinformatics analysis of the 59 ORF harbored in SRL PAI was performed (Table 2). A previous bioinformatics analysis was conducted by Luck and coworkers [6] (Table 2, left side), however an updated analysis of each ORF showed significant modifications due to the updated information available in recent data repository (NCBI). Forty percent of the proteins were re-assigned with significant differences (Table 2). From our analysis, *orf8* product was selected as the most probable contributor to the phenotype associated to D-Asp as an aspartate racemase (Table 2). Interestingly *orf9*, has been identified as an anaerobic C4-dicarboxylate membrane transporter protein DcuA (100% identity, Table 2) and it also could be associated with D/L-aspartate translocation through the cell. Therefore, we selected *orf8-orf9* region from the SRL PAI (S1 Fig) as the probable genes responsible for the observed phenotype. In parallel, the role of the island in the metabolism of other phenotypes identified by PMs, such as D-raffinose, was assessed, however, no evident connection between them was found. It is possible that these phenotypes are related to the interaction of the SRL island with metabolic routes encoded by other sequences in the genome. Further studies are needed in order to determine their molecular cause.

**Table 2:** Update of the probable products of the ORFs contained in the SRL PAI.

ORF^1^	Gene	Related protein (Luck *et al*., 2001*)*	% Similarity^#^	Related protein (This work)	% Similarity^#^
2	*orf2*	Yfjl	45	Hypothetical protein (DUF 3987)	100*
4	*orf4*	No similarity	-	Hypothetical protein	100*
5	*orf5*	No similarity	-	Inovirus Gp2 family protein	100*
6	*orf6*	Yfjl (frameshift)	73	Inovirus Gp2 family protein	100
7	*orf7*	LysR-like transcriptional regulator	79	LysR-like transcriptional regulator	100
8	*orf8*	Hypothetical protein in LysR-AraE	63	Aspartate racemase	99.56
9	*orf9*	DcuA (anaerobic decarboxylate transporter)	70	DcuA (anaerobic decarboxylate transporter)	100*
17	*orf17*	YdjB	79	Amino acid-binding protein	100 *
18	*orf18*	JemC	79	ArsR family transcriptional regulator	95.88
23	*orf23*	CapU (hexosyltransferase homolog)	99, 73	Glycosyl transferase family 1	100 *
24	*orf24*	Shf	93, 92.3	Putative protein shf	100+
25	*orf25*	Hypothetical ORF o137	98	Hypothetical protein	100
34	*orf34*	Putative periplasmic protein (*Campylobacter jejuni*)	52	Esterase-like activity of phytase family protein	99.78
35	*orf35*	L0015	98	IS66 family transposase	99.57
36	*orf36*	L0014	98	IS66 family insertion sequence element accessory protein TnpB	100*
37	*orf37*	L0013	99	Transposase	99.25
38	*orf38*	No similarity	-	Hypothetical protein	100*
39	*orf39*	YfjJ	55	Inovirus Gp2 family protein	100*
40	*orf40*	Hha/YmoA (*Yersinia enterocolitica*)	75, 75	Hemolysin activation protein	100
41	*orf41*	Vis (P4)	53	AlpA family transcriptional regulator	100*
42	*orf42*	No similarity	-	Hypothetical protein	100*
43	*orf43*	No similarity	-	Uncharacterized protein	75.52
44	*orf44*	No similarity	-	Hypothetical protein	100*
45	*orf45*	No similarity	-	Hypothetical protein	100*
46	*orf46*	YfjP, YeeP	65, 97	50S ribosome-binding GTPase	99.66
47	*orf47*	Ag43 (Flu)	78	autotransporter adhesin Ag43	99.47+
48	*orf48*	YfjQ	88	DUF945 domain-containing protein, partial	98.86
49	*orf49*	YfjX, KlcA	81, 51	Hypothetical protein SFxv_1159	93.33 +
50	*orf50*	YfjY, YeeS	80, 99	Putative radC-like protein yeeS	98.77
51	*orf51*	YeeT	98	DNA repair RadC family protein, partial	100
52	*orf52*	YeeU, YfjZ	91, 75	type IV toxin-antitoxin system YeeU family antitoxin	97.58*
53	*orf53*	L0007, YeeV, YpjJ	93, 87, 74	Toxin	100 *
54	*orf54*	L0008, YeeW	96, 77	hypothetical protein	99.39*
55	*orf55*	L0009	88	DUF957 domain-containing protein	100*
56	*orf56*	L0010	85	DUF4942 domain-containing protein	100
57	*orf57*	L0011	80	hypothetical protein ESMG_02720	91.04
58	*orf58*	IS/328 transposase (*Y*. *enterocolitica*)	83	IS110-like element ISSfl8 family transposase	100 *
59	*orf59*	L0012	90	hypothetical protein G960_01162	98.72

^1^ORFs contained in the SRL pathogenicity island excluding *orf1* (integrase), *orf3* (*rox*), *orf10*-*orf17*/*orf19-orf22* (antibiotic resistance genes), and *orf26*-*orf33* (Ferric Dicitrate Transport System), which have known protein products.

^#^Related proteins are from *E*. *coli* unless otherwise stated.

*Related to Multispecies proteins [*Enterobacteriaceae*] or [Gammaproteobacteria]

+Related to *S*. *flexneri* strain 2a

**Fig 2:**
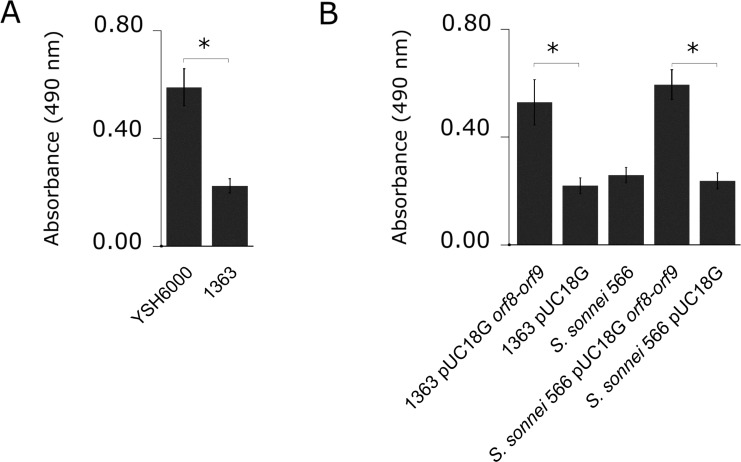
*orf8-orf9* region is responsible for metabolization of D-aspartic acid as a sole carbon source. *Shigella flexneri* and *Shigella sonnei* strains were cultured on S*higella* Minimal Medium supplemented with 0.01% w/v TTC as a redox indicator. (A) YSH6000 and its isogenic mutant for the SRL PAI, 1363 strain are shown. (B) *S*. *flexneri* and *S*. *sonnei* SRL PAI-negative strains complemented with the *orf8-orf9* region and their controls are shown. The graph shows the absorbance at 490 nm of the bacterial cultures at 24 h (final absorbance minus initial absorbance). ANOVA and Tukey tests were performed, and significant results are represented by asterisks (* = *p*<0.05). Error bars represent standard deviation.

To assess the role of the putative aspartate racemase and the DcuA protein in bacterial respiration in presence of D-Asp, the *orf8-orf9* region including its putative promoter upstream of *orf8*, was cloned within the pUC18G vector, originating pUC18G *orf8-orf9*. Negative strains for the SRL PAI, *S*. *flexneri* 2a 1363 and *S*. *sonnei* 566, were independently transformed with the recombinant plasmid and with pUC18G empty vector (as control). *S*. *sonnei* was used as an additional negative control, being a related phylogenetic strain not harboring the SRL PAI. The phenotypes were analyzed by culturing *Shigella* strains in minimal medium with D-Asp as a sole carbon source and 0.01% TTC as a redox indicator. Using these conditions, *S*. *flexneri* 2a YSH6000 was able to metabolize D-Asp to a significantly higher extent than *S*. *flexneri* 1363, in accordance to the results from the Phenotype MicroArrays (Fig 2A). Moreover, negative SRL PAI strains (both *S*. *flexneri* 2a and *S*. *sonnei*), transformed with the o*rf8-orf9* region acquired the phenotype associated to D-Asp metabolism (Fig 2B). Control experiments transforming *S*. *flexneri* 1363 and *S*. *sonnei* 566 with pUC18G empty plasmid showed that this vector by itself was not able to modify this phenotype (Fig 2B).

### Racemization of D-aspartate requires *orf8-orf9* region

**Fig 3:**
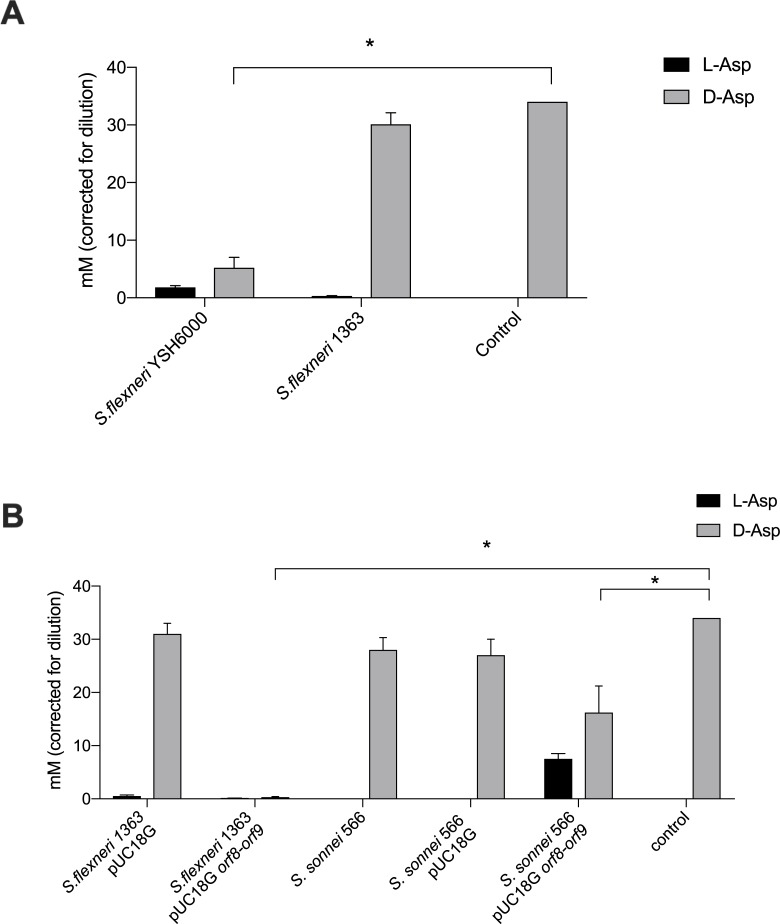
Quantification of D- and L- form of aspartic acid in cultures supernatants using HPLC-MS/MS. Aspartic acid isomers were measured in supernatants of cultures in minimal medium after 24 h of incubation at 37ºC for strains YSH6000 and its isogenic mutant, 1363 (A) and for *S*. *flexneri* and *S*. *sonnei* SRL PAI-negative strains complemented with the *orf8-orf9* region (B). ANOVA and Tukey tests were performed and significant results are represented by asterisks (* = *p*<0.05). Error bars represent standard errors.

We measured to what extent *orf8-orf9* region (aspartate racemase and its putative transporter) was involved in racemization of D- and L- form of aspartic acid. Minimal medium enriched with 30 mM of D-Asp was incubated with wild type and mutant strains (10^8^ CFU/mL) and the racemization activity was measured via HPLC/MS-MS. As a control, minimal medium with D-Asp 30 mM without bacteria was used. Results showed that the wild type strain *S*. *flexneri* YSH6000 significantly metabolized the D-form compared to the control (Fig 3A). Also, we found that the level of D-Asp decreased in the supernatant of the *S*. *flexneri* YSH6000 strain, but also a moderate accumulation of the L- form was found as result of the racemization reaction (Fig 3A). On the other hand, the *S*. *flexneri* 1363 strain was not able to metabolize D-Asp, as concentration remained similar to the control medium (medium without bacteria) (Fig 3B). *S*. *flexneri* 1363 complemented with the *orf8-orf9* region (1363 pUC18G *orf8-orf9*), on the opposite, showed almost a total reduction of the D-form. The additional control, *S*. *sonnei* 566 strain complemented (566 pUC18G *orf8-orf9*) also showed a significant decrease of D-Asp and a concomitant increase of the L- form due to racemization (Fig 3B). This result supports the sufficiency of the region to produce the phenotype independently of the genetic background.

### Growth in D-Asp as carbon source requires both *orf8-orf9*

**Fig 4:**
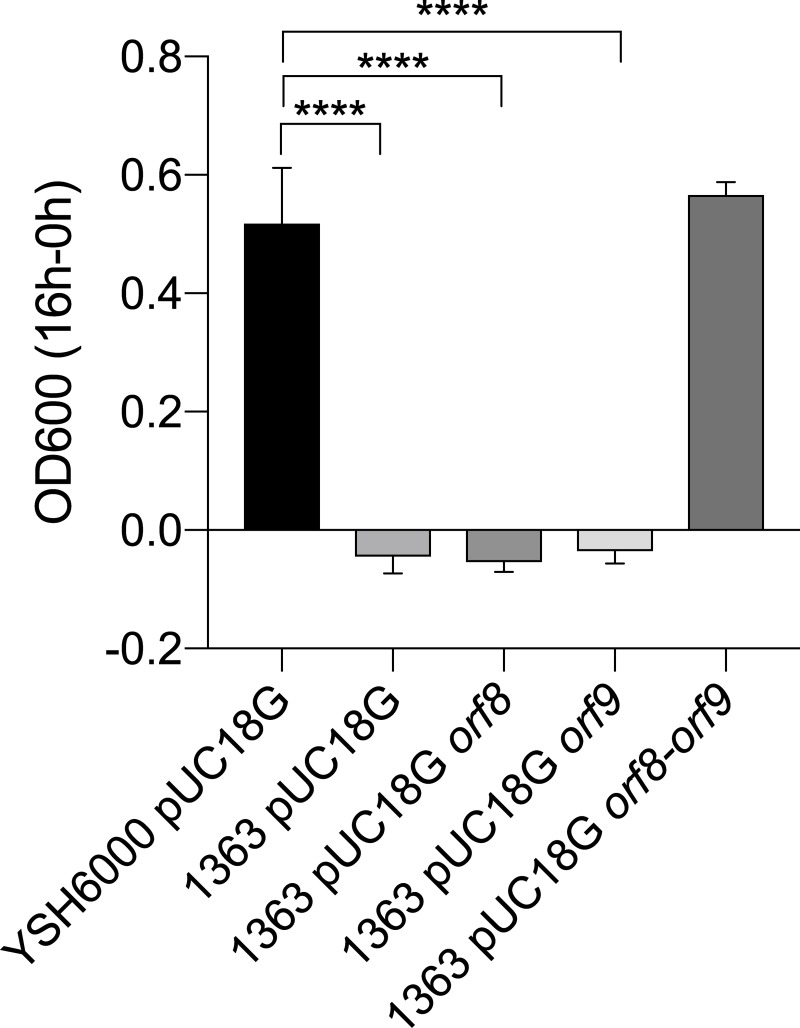
Bacterial growth in minimal medium with D-Asp as sole carbon source after 16 hours of incubation. Absorbance at 600 nm was measured after overnight incubation (16 hours) at 37°C in optimized minimal medium with D-Asp and used to calculate final minus initial OD600. ANOVA test was performed, and significant results are represented by asterisks (**** = *p*<0.0001). Error bars represent standard deviation.

In order to determine whether the metabolism of D-Asp was related to the individual activity of the product of *orf8* (racemase) or *orf9* (transporter), each gene was cloned into the modified pUC18G and used to transform *S*. *flexneri* 2a 1363. In parallel, an optimized minimal medium with D-Asp to support growth in presence of 2 μg mL^-1^ gentamicin was generated and YSH6000 transformed with the empty vector (YSH6000 pUC18G) was used as control. After 16 h of incubation at 37°C, our results indicated that *S*. *flexneri* 2a 1363 complemented with the *orf8-orf9* region is able to grow in presence of D-Asp as sole carbon source, similarly to the reference strain YSH6000 (Fig 4). However, the strains transformed with individual genes (1363 pUC18G *orf8* and 1363 pUC18G *orf9*) could not grow in presence of D-Asp. Indeed, the data indicated that the OD of these strains and the 1363 pUC18G (SRL PAI negative) decreased after 16 hours in comparison to the initial inoculum, which supports the toxic effect of D-Asp in absence of the *orf8-orf9* region (Fig 4).

Further, to analyze the growth dynamics of the strains in presence of D-Asp, growth curves using optimized minimal medium with D-Asp or L-Asp (as comparison) were performed. Our results indicated that all the strains are able to use L-Asp as sole carbon source (S2 Fig). However, in presence of D-Asp as carbon source, only the strain positive for the SRL island (YSH6000 pUC18G) and the 1363 strain complemented with the *orf8-orf9* region (1363 pUC18G *orf8-orf9*) grew, similarly to what was observed in the overnight experiment (S2 Fig). These results support the protective role of the *orf8-orf9* from the SRL island against the toxicity of D-Asp and the relevance of this region for the metabolism of this enantiomer as carbon source.

## Discussion

*S*. *flexneri* is an enteropathogen globally isolated from different environments such as water, soil and food [9–10]. In this context, the characterization of the ecology of *Shigella* in its natural niches is important to understand the life cycle outside the human body, its natural reservoir. In certain strains of *S*. *flexneri*, mobile genetic elements like pathogenicity islands are responsible for the resistance to some toxic compounds found in the environment, such as antibiotics [21]. SRL PAI of *S*. *flexneri* 2a YSH6000 carries 59 ORFs and the contribution of these ORFs to the fitness and response to the environmental changes is still unknown. In this work we have shown that the SRL PAI allows the metabolization of D-Asp via racemization. A bioinformatics analysis identified the *orf8* as an aspartate racemase, and *orf9* as an anaerobic C4-dicarboxylate membrane transporter protein, DcuA, possibly involved in the aspartic acid translocation [22]. D-aspartic acid is widely available in the environment and it is a toxic compound that acts as an inhibitor of the synthesis of proteins in bacteria [19, 20]. Indeed, Eisenstadt and coworkers described in 1959 that *Pseudomonas saccharophila* (*Pelomonas saccharophila*) was unable to grow in presence of D-Asp when added to a minimal medium enriched with maltose or cellobiose. The authors described that this phenomenon was only visible using some carbon sources and suggested that these variations could be associated to differences in the levels of L-aspartate in the internal free amino acid pool of cells grown on the different carbon sources. Similarly, the toxic effect of 0.01% D-Asp over bacterial growth could be seen in our results in minimal medium with mannose as a carbon source and not in mannitol (the two carbon sources metabolized by these *Shigella* strains, according to Vitek results). However, the YSH6000 strain seems to be able to metabolize it. Our results of growing bacteria in mannose and mannitol also agree with the work of Eisenstadt and coworkers about the toxic effect of D-Asp dependent on the type of carbon source and internal L-aspartate pool. In this context, recent works have explored the role of racemases and other enzymes in the metabolization of D-amino acids. For example, racemic amino acids are present in the environment and resident bacteria consume D- and L- enantiomers, either simultaneously or sequentially depending on the level of their racemase activity [16]. Bacteria thus protect life on earth by keeping D-amino acid free environment [16]. Similarly, amino acid malonylation in plants has been proposed as a mechanism to detoxify these compounds [23]. Thus, it is possible to suggest that the presence of the aspartate racemase in the SRL PAI, and therefore, the ability to perform racemization of D-Asp would be beneficial to cope with the high concentrations of D-amino acids in soil and plants. Indeed, our preliminary results indicated that *S*. *flexneri* 2a YSH6000 is able to persist in significantly higher amounts in tomato pericarp in comparison to the *S*. *flexneri* 2a 1363 (data not shown).

Interestingly, it was recently described that bacterial D-amino acids and racemases have a role in signaling in the human gut [24–25]. Similarly, another publication described the presence of 12 free D-amino acids (including D-Asp) in the intestine. According to the authors, these molecules are produced mostly by bacteria belonging to the phylum Firmicutes and could have a role in bacterial communication [26]. These findings lead us to wonder if this aspartate racemase could be involved in the colonization of the human gastrointestinal tract. Indeed, some racemases have been already linked to survival and pathogenesis, such as in the case of an alanine racemase in *Salmonella enterica* serovar Enteritidis [27].

The ability to metabolize D-Asp as a carbon source has been previously described in environmental strains [28, 29]. Protein similarity sequences analysis of the products of *orf8-orf9* region from *S*. *flexneri* YSH6000 showed that it is also present as a functional unit in *E*. *coli* strains such as *E*. *coli* HUSEC2011, *E*. *coli* O104:H4 str. 2009EL-2071 and *E*. *coli* O104:H4 str. 2011C-3493, suggesting that racemization of D-amino acids would occur in other pathogenic organisms.

From the ecological point of view, pathogenicity islands such as SRL PAI are tools for horizontal gene transfer [30] and by extension we speculate that these genes get a benefit by staying within the island.

## Conclusion

Our results indicate that both aspartate racemase and the transporter encoded by the *orf8-orf9* region of the SRL PAI allows *Shigella* to metabolize D-Asp, conferring an advantage using this D-amino acid as carbon source or detoxifying its toxic effect. The study of the common heritance of these genes in enterobacteria and other bacterial strains is therefore important to understand the relevance of this strategy for bacterial adaptation to the environment and pathogenicity.

## Supporting information

S1 Fig Genetic organization of *orf8-orf9* region in the SRL pathogenicity island. The arrows represent the coding regions: *orf8* is shown in blue and *orf9* in green. Black circles show the regions were putative promoters identified by bioinformatic analysis were found (BPROM-Softberry).
(EPS)

S2 Fig Growth dynamics in minimal medium with D-Asp or L-Asp as sole carbon source. Growth curves in optimized minimal medium in presence of D-Asp or L-Asp as sole carbon source were performed at 37° with continuous shaking. For each point, 0.5 ml of culture was taken and measured at 600 nm.
(EPS)

S1 Table Table of strains and plasmids used in this study. (DOCX)

S2 Table Table of Vitek Compact 2 biochemical characterization of reference strains. (DOCX)

S3 Table Data table obtained by Phenotype Microarray analysis (Biolog) for the reference strains. The results show the average measurement for each strain (YSH6000 and 1363) through the 48 hours of the first and second run of the experiment.
(XLS)
