# Supplementary material for: SRL pathogenicity island contributes to the metabolism of D-aspartate via an aspartate racemase in Shigella flexneri YSH6000
Source: PLoS One. 2020 Jan 24;15(1):e0228178. doi: 10.1371/journal.pone.0228178 (PMC6980539; doi:10.1371/journal.pone.0228178)
Supplement: S1 Table — (DOCX) [file pone.0228178.s003.docx]

**S1 Table. Strains and plasmids used in this study**

| **Strain name** | **Species** | **Relevant characteristics** | **Reference** |
| --- | --- | --- | --- |
| YSH6000 | *S. flexneri* | Wild type for the SRL island | Luck et al., 2001 |
| 1363 | *S. flexneri* | Spontaneous SRL PAI deletant of YSH6000 | Turner et al., 2001  (SBA 1363) |
| *S. sonnei* 566 | *S. sonnei* | Wild type strain negative for the SRL island | This study |
|  |  | |  |
| **Plasmid name** | **Relevant characteristics** | | **Reference** |
| pUC18G | pUC18 plasmid with gentamicin cassette in SspI site | | This study |
| pUC18G *orf8-orf9* | pUC18 plasmid with gentamicin cassette in SspI site and *orf8-orf9* region cloned in MCS | | This study |
| pUC18G *orf8* | pUC18 plasmid with gentamicin cassette in SspI site and *orf8* region cloned in MCS | | This study |
| pUC18G *orf9* | pUC18 plasmid with gentamicin cassette in SspI site and *orf9* region cloned in MCS | | This study |
