# Supplementary material for: SRL pathogenicity island contributes to the metabolism of D-aspartate via an aspartate racemase in Shigella flexneri YSH6000
Source: PLoS One. 2020 Jan 24;15(1):e0228178. doi: 10.1371/journal.pone.0228178 (PMC6980539; doi:10.1371/journal.pone.0228178)
Supplement: S2 Table — (DOCX) [file pone.0228178.s004.docx]

| **Test** | **YSH6000** | **1363** |
| --- | --- | --- |
| Ala-Phe-Pro-ARYLAMIDASE | - | - |
| ADONITOL | - | - |
| L-Pyrrolydonyl-ARYLAMIDASE | - | - |
| L-ARABITOL | - | - |
| D-CELLOBIOSE | - | - |
| BETA-GALACTOSIDASE | - | - |
| H2S PRODUCTION | - | - |
| BETA-N-ACETYL-GLUCOSAMINIDASE | - | - |
| Glutamyl Arylamidase pNA | - | - |
| D-GLUCOSE | + | + |
| GAMMA-GLUTAMYL-TRANSFERASE | - | - |
| FERMENTATION/ GLUCOSE | + | + |
| BETA-GLUCOSIDASE | - | - |
| D-MALTOSE | - | - |
| D-MANNITOL | + | + |
| D-MANNOSE | + | + |
| BETA-XYLOSIDASE | - | - |
| BETA-Alanine arylamidase pNA | - | - |
| L-Proline ARYLAMIDASE | - | - |
| LIPASE | - | - |
| PALATINOSE | - | - |
| Tyrosine ARYLAMIDASE | - | - |
| UREASE | - | - |
| D-SORBITOL | - | - |
| SACCHAROSE/SUCROSE | - | - |
| D-TAGATOSE | - | - |
| D-TREHALOSE | + | + |
| CITRATE (SODIUM) | - | - |
| MALONATE | - | - |
| 5-KETO-D-GLUCONATE | - | - |
| L-LACTATE alkalinization | - | - |
| ALPHA-GLUCOSIDASE | - | - |
| SUCCINATE alkalinization | - | - |
| Beta-N-ACETYL-GALACTOSAMINIDASE | - | - |
| ALPHA-GALACTOSIDASE | - | - |
| PHOSPHATASE | + | + |
| Glycine ARYLAMIDASE | - | - |
| ORNITHINE DECARBOXYLASE | - | - |
| LYSINE DECARBOXYLASE | - | - |
| L-HISTIDINE assimilation | - | - |
| COUMARATE | + | + |
| BETA-GLUCURONIDASE | - | - |
| O/129 RESISTANCE (comp.vibrio.) | + | + |
| Glu-Gly-Arg-ARYLAMIDASE | - | - |
| L-MALATE assimilation | - | - |
| ELLMAN | - | - |
| L-LACTATE assimilation | - | - |

**S2 Table. Biochemical characterization of strains YSH6000 and 1363 by Vitek Compact 2.**
